# β-Glucuronidase Pattern Predicted From Gut Metagenomes Indicates Potentially Diversified Pharmacomicrobiomics

**DOI:** 10.3389/fmicb.2022.826994

**Published:** 2022-03-03

**Authors:** Francesco Candeliere, Stefano Raimondi, Raffaella Ranieri, Eliana Musmeci, Alfonso Zambon, Alberto Amaretti, Maddalena Rossi

**Affiliations:** ^1^Department of Life Sciences, University of Modena and Reggio Emilia, Modena, Italy; ^2^Department of Chemistry and Geological Sciences, University of Modena and Reggio Emilia, Modena, Italy; ^3^Biogest-Siteia, University of Modena and Reggio Emilia, Modena, Italy

**Keywords:** β-glucuronidase, human gut microbiota, metagenome, WGS, whole genome sequencing, drug metabolism, pharmacomicrobiomics

## Abstract

β-glucuronidases (GUS) of intestinal bacteria remove glucuronic acid from glucoronides, reversing phase II metabolism of the liver and affecting the level of active deconjugated metabolites deriving from drugs or xenobiotics. Two hundred seventy-nine non-redundant GUS sequences are known in the gut microbiota, classified in seven structural categories (NL, L1, L2, mL1, mL2, mL1,2, and NC) with different biocatalytic properties. In the present study, the intestinal metagenome of 60 healthy subjects from five geographically different cohorts was assembled, binned, and mined to determine qualitative and quantitative differences in GUS profile, potentially affecting response to drugs and xenobiotics. Each metagenome harbored 4–70 different GUS, altogether accounting for 218. The amount of intestinal bacteria with at least one GUS gene was highly variable, from 0.7 to 82.2%, 25.7% on average. No significant difference among cohorts could be identified, except for the Ethiopia (ETH) cohort where GUS-encoding bacteria were significantly less abundant. The structural categories were differently distributed among the metagenomes, but without any statistical significance related to the cohorts. GUS profiles were generally dominated by the category NL, followed by mL1, L2, and L1. The GUS categories most involved in the hydrolysis of small molecules, including drugs, are L1 and mL1. Bacteria contributing to these categories belonged to *Bacteroides ovatus*, *Bacteroides dorei*, *Bacteroides fragilis*, *Escherichia coli*, *Eubacterium eligens*, *Faecalibacterium prausnitzii*, *Parabacteroides merdae*, and *Ruminococcus gnavus*. Bacteria harboring L1 GUS were generally scarcely abundant (<1.3%), except in three metagenomes, where they reached up to 24.3% for the contribution of *E. coli* and *F. prausnitzii.* Bacteria harboring mL1 GUS were significantly more abundant (mean = 4.6%), with *Bacteroides* representing a major contributor. Albeit mL1 enzymes are less active than L1 ones, *Bacteroides* likely plays a pivotal role in the deglucuronidation, due to its remarkable abundance in the microbiomes. The observed broad interindividual heterogeneity of GUS profiles, particularly of the L1 and mL1 categories, likely represent a major driver of pharmacomicrobiomics variability, affecting drug response and toxicity. Different geographical origins, genetic, nutritional, and lifestyle features of the hosts seemed not to be relevant in the definition of glucuronidase activity, albeit they influenced the richness of the GUS profile.

## Introduction

Humans and their colon microbiota evolved together, establishing a close symbiotic interrelationship, fruitful for both. The gut microbiota is implicated in a number of biological processes such as resistance to colonization (Ruan et al., 2020), immune system modulation (Saldana-Morales et al., 2021), synthesis of essential vitamins and nutrients (Oliphant and Allen-Vercoe, 2019), and breakdown of undigested polysaccharides and proteins (El Kaoutari et al., 2013; Huang et al., 2017; Raimondi et al., 2021). Furthermore, it encodes a broad diversity of enzymes capable of processing foreign compounds (e.g., phytochemicals, environmental pollutants, pharmaceuticals, and other xenobiotics) and their endogenous metabolites, adding significant chemical diversity and modifying lifetimes, bioavailability, and biological activity (Rossi et al., 2013; Koppel et al., 2017). In this context, pharmacomicrobiomics is an emerging field focusing on the interplay of microbiome and drug metabolism and response (Doestzada et al., 2018; Hassan et al., 2021).

Hundreds of bacterial enzymes are dedicated to the hydrolysis of carbohydrates and glycoconjugates that are not digested in the upper gut and reach the colon, where they are broken down by the microbiota (Flint et al., 2012). Among these enzymes, β-glucuronidases (GUS) remove glucuronic acid from glucoronides, reversing the phase II metabolism carried out by liver enzymes on endo- and xeno-biotics in order to facilitate their excretion from the body (Ervin and Redinbo, 2020). Glucuronic acid is then utilized by bacteria as a carbon and energy source, being channeled into the Entner–Doudoroff pathway that catabolizes sugar acids into pyruvate (Peekhaus and Conway, 1998). The deglucuronidated compounds can be reabsorbed through the gut epithelium and reach the plasma, in a process called enterohepatic circulation (Roberts et al., 2002; Pellock and Redinbo, 2017). Thus, bacterial GUS affect the pharmacokinetics of compounds such as polyphenols, xenobiotics, and drugs and participate in the regulation of the levels of circulating metabolites, altering the pharmacological properties and the biological activities of xenobiotics and potentially impacting on their beneficial and/or toxic effects on health (Biernat et al., 2018; Wang et al., 2019; Awolade et al., 2020).

GUS were first identified in 1934 in *Escherichia coli* and other Enterobacteriaceae (Masamune, 1934; Oshima, 1934), but later, they have been detected in several bacterial taxa belonging to all the main phyla within the gut microbiota: Bacteroidetes, Firmicutes, Proteobacteria, and Actinobacteria (McBain and Macfarlane, 1998; Russell and Klaenhammer, 2001; Nakamura et al., 2002; Gloux et al., 2011). Nowadays, it is known that intestinal bacteria encode different GUS types with structural differences affecting function, biocatalytic properties, and substrate specificity (Biernat et al., 2019; Parvez et al., 2021). The driving force for such evolution and diversification of bacterial GUS has been the availability of dietary and endogenous glucuronides to the commensal microbiota (Pellock and Redinbo, 2017). In particular, the glucuronides of several endogenous metabolites (such as bilirubin, estrogen and androgen hormones, neurotransmitters, and bile acids) are produced by liver UDP-glucuronosyltransferase and abundantly excreted into the intestinal lumen (Liston et al., 2001; Meech et al., 2012; Jarrar and Lee, 2021). The massive sequencing of the human intestinal metagenomes in the Human Microbiome Project (HMP) (Turnbaugh et al., 2007) and bioinformatic mining tools enabled the identification of a wide repertoire of GUS encoded by human gut bacteria. The so-called GUSome has been proposed, encompassing 279 non-redundant GUS sequences (Pollet et al., 2017), 93.5% of which have been taxonomically assigned to Bacteroidetes (52%), Firmicutes (43%), Verrucomicrobia (1.5%), and Proteobacteria (0.5%) (Pollet et al., 2017).

Bacterial GUS present a conserved folding, with two structural elements (loop 1 and loop 2), adjacent to the active site, that differ in length and amino acid composition and permit classification into seven GUS structural categories: NL, L1, L2, mL1, mL2, mL1,2, and NC (Pollet et al., 2017). The enzymes of diverse categories differ in size, substrate-binding modules, active site features, and subcellular localization. Most of the intestinal GUS belong to the category NL (57.3%), followed by mL1, L2, L1, mL2, NC, and mL1,2 in decreasing order (Pollet et al., 2017). The dimension of the loops is pivotal for substrate recognition and affects the biocatalytic properties of the enzymes. Categories L1, mL1, and L2 are more efficient to catalyze the deglucuronidation of small substrates in comparison to categories mL2, mL1,2, and NL (Wallace et al., 2015; Biernat et al., 2019). Differences in the cellular localization are related to the category: L1 enzymes lack signal peptide and are intracellular, whereas L2, mL2, and mL1,2 GUS are likely extracellular. For the GUS belonging to categories mL1 and NL, the presence of signal peptide is linked to the phylum: absent in Firmicutes and present in Bacteroidetes.

The microbial composition of intestinal microbiota impacts GUS abundance and diversity, with major effects on the metabolism of drugs and xenobiotics likely responsible for different individual responses (Elmassry et al., 2021). This study wanted to determine the qualitative and quantitative differences of GUS-encoding genes among metagenomes of healthy subjects. It aimed to investigate the interindividual variability of GUS-encoding bacteria in the gut, mining 60 intestinal publicly available metagenomes of healthy subjects. To circumvent the bias arising from diverse genetic, nutritional, and lifestyle features, the metagenomes belonging to five geographically different cohorts were retrieved and processed for GUS profiling. This approach provided preliminary information of interindividual differences of the GUS repertoire, with awareness that transformation of drugs and xenobiotics is subjected to regulation of the expression. The results herein presented could promote intentional manipulation of gut microbiota to enhance drug effectiveness in order to reduce adverse drug interactions or other approaches of personalized therapy to obtain maximum efficacy and minimum toxicity.

## Materials and Methods

### Metagenomes

Sixty publicly available metagenomes of gut microbiota from healthy adults were collected from the NCBI Sequence Read Archive (SRA), with the accession numbers listed in Supplementary Table 1. The subjects were ascribed to five cohorts from five different countries: China (CHN), Ethiopia (ETH), Spain (ESP), United States of America (USA), and Sweden (SWE). The selected metagenomes were sequenced through whole-genome shotgun sequencing on Illumina paired-end platforms and produced reads ranging between 100 and 150 bp in length.

### Assembly and Binning

The FASTQ files were checked for quality and primer presence with FastQC v0.11.8 (Andrews, 2010), in order to assure that only high-quality reads (length > 50 bp; quality score > 20) were further analyzed. When necessary, the tool Cutadapt v1.16 (minimum length 50; quality cutoff 20) (Martin, 2011) was used for quality filtering. The cohort ESP required primer removal, which was carried out through Trimmomatic (Bolger et al., 2014) with ILLUMINACLIP setting. The reads were assembled in contigs using metaSPAdes v 3.9 (Nurk et al., 2017) with default parameters. The contigs were binned with MaxBin2 v2.2.7 (Wu et al., 2016) to obtain metagenome-assembled genomes (MAGs). MaxBin2 measures the tetranucleotide frequencies of the contigs and their coverages to classify them into individual bins. It employs single-copy marker gene prediction to determine the completeness of bins (Wu et al., 2014, 2016). According to MaxBin2 default parameters, only contigs at least 1,000 bp long were utilized for binning, and those shorter were discarded from further analysis. MAGs were taxonomically identified with the CAT/BAT tool (von Meijenfeldt et al., 2019). Each bin was mapped against the raw reads using Bowtie2 (Langmead and Salzberg, 2013) to assess the relative abundance. Except for CAT/BAT that was run locally, the steps were conducted on Galaxy platform^1^ (Afgan et al., 2018).

## Bacterial Composition and Alpha and Beta Diversity

The relative abundance of taxonomically identified MAGs was used to define the abundance profile of bacterial taxa in each metagenome. A BIOM file was produced and imported into Qiime2 (Bolyen et al., 2019) to compute beta diversity according to Bray–Curtis dissimilarity. The beta distance matrix was utilized for principal coordinate analysis (PCoA). Bacterial composition at a deeper taxonomic level was assessed by MetaPhLan2 (Segata et al., 2012; Truong et al., 2015) for the species *Clostridium perfringens*, *Eubacterium eligens*, *Lactobacillus rhamnosus*, *Ruminococcus gnavus*, *Streptococcus agalactiae*, *Bacteroides uniformis*, *Bacteroides ovatus*, *Bacteroides dorei*, *Bacteroides fragilis*, and *Parabacteroides merdae*, known to encode several deeply characterized GUS (Pellock et al., 2018; Biernat et al., 2019; Ervin et al., 2019). Alpha diversity has been calculated using Shannon index, Chao-1 index, and Pielou’s evenness with the tool Past v 4.08 (Hammer et al., 2001).

### β-Glucuronidase Identification and Profiling

The 279 sequences of GUS identified and classified by Pollet et al. (2017), listed in Supplementary Material 1, were blasted to the binned metagenomes using tBLASTn with an *e*-value 10^–100^ (Altschul et al., 1990). The results were filtered at a high identity percentage (≥98.5%). Redundant hits mapping on the same position of the same contig were discarded.

The abundance of each GUS was correlated to the abundance of the bin containing the contig where the GUS sequence was mapped to. In particular, the abundance of each GUS was calculated taking into account the number of reads mapping on the corresponding bin. The Jaccard similarity was computed to estimate the beta diversity based on GUS profiles and subjected to PCoA.

### Statistical Analysis

Statistical analysis using ANOVA (*p* < 0.05) followed by Tukey’s *post hoc* test was conducted to compare cohorts in terms of GUS profiles, abundance of bacteria harboring GUS genes, and relative abundance of each GUS structural category. Alpha diversity indices of cohorts were compared with the Kruskal–Wallis test followed by Dunn’s multiple-comparison test. In beta diversity analysis of microbiome composition and GUS profile, the statistical significance among cohorts was analyzed with PERMANOVA statistical test (*p* < 0.05).

## Results

### Metagenomic Analysis

**FIGURE 1:**
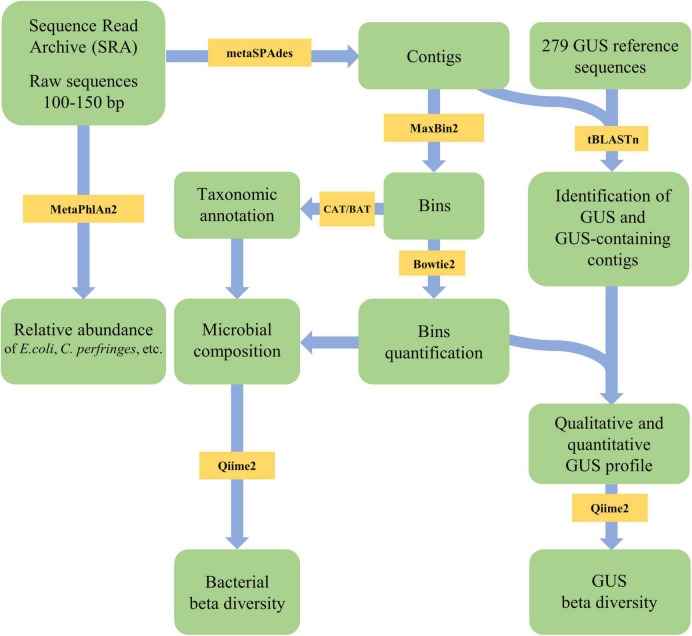
Flowsheet of the pipeline applied in the present study for metagenome analysis and β-glucuronidases (GUS) search.

Sixty metagenomes of gut microbiota from healthy subjects, sequenced with Illumina paired-end technology, were retrieved and scanned according to the flowsheet reported in Figure 1 to search the genes encoding the 279 GUS proteins identified by Pollet et al. (2017). The metagenomes encompassed 44 ± 35 million reads (mean ± SD), with lengths ranging between 100 and 150 bp (Supplementary Figure 1A). Assembly of metagenomes with MetaSPAdes yielded on average 408,745 ± 131,363 contigs per metagenome (mean ± SD) (Supplementary Figure 1B).

Reference-free binning with MaxBin2 recovered from 29 to 179 binned genomes per subject, with a mean value of 72 (Supplementary Figure 1C). For most metagenomes (>75%), the reads associated with a bin, mapped by Bowtie2, accounted for more than 80% (mean 85.2%) (Supplementary Figure 1D).

### Bacterial Composition and Beta Diversity

**FIGURE 2:**
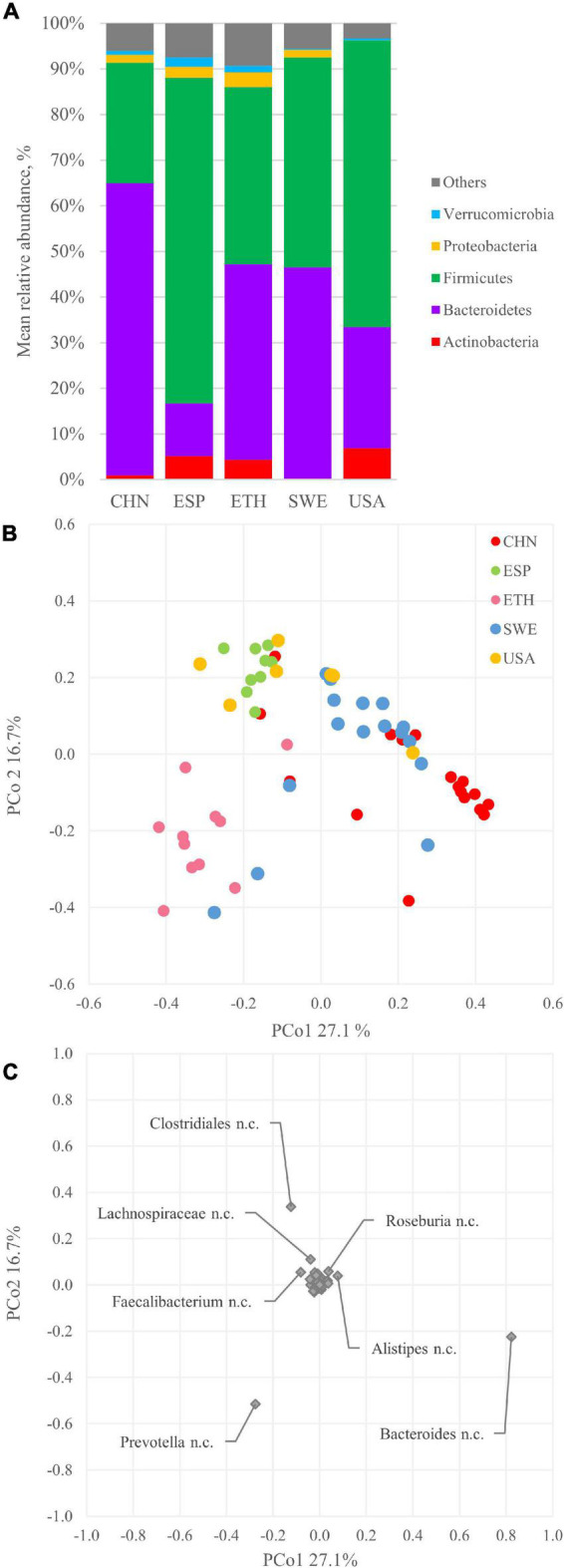
Bacterial composition and beta diversity of gut metagenomes of 60 healthy adults. **(A)** Mean relative abundance of the main phyla identified in the whole dataset and in the five cohorts. **(B)** principal coordinate analysis (PCoA) plot of beta diversity based on Bray–Curtis dissimilarity index of the microbial composition. **(C)** PCoA plot of the species contribution to metagenome differentiation.

The bins were quantified with Bowtie2 and assigned a taxonomic designation with CAT/BAT. The dominant phyla were Firmicutes and Bacteroidetes, with the former generally outnumbering the others and the latter dominating only CHN metagenomes (Figure 2A). The relative amounts of Actinobacteria and Proteobacteria were quite different among subjects, lying in the range of 0.2–6.9% and 0.1–3.3%, respectively. Verrucomicrobia ranged from 0.2 to 2.0% of the whole bacterial population. Bins ascribed to other phyla or lacking taxonomic attribution (labeled as “others”) ranged from 3.3 to 9.3%. At deeper taxonomic level, the quantity of unclassified bins increased; thus, the profiling was less accurate. Among families, Bacteroidaceae were among the most abundant, with a mean of 22.5% in 60 metagenomes, resulting in its prevalence in the CHN cohort. Prevotellaceae were remarkably higher in the ETH cohort compared to the others. A similar distribution was observed in genera distribution, with *Bacteroides* prevailing in the CHN cohort and *Prevotella* in the ETH cohort (Supplementary Figure 2). For each cohort, core genera present in at least 85% of subjects were identified. Genera *Alistipes*, *Bacteroides*, *Faecalibacterium*, and *Ruminococcus* were identified in all the cohort (Supplementary Figure 3).

Alpha diversity of metagenomes was evaluated with Shannon index, Chao-1 index, and Pielou’s evenness (Supplementary Figure 4). Shannon index showed a significant difference (*p* < 0.05) between the CHN and ETH cohorts. Chao-1 richness highlighted higher values for the SWE cohort, significantly different from the CHN and USA cohorts. Pielou’s evenness presented high values for all cohorts, with CHN showing a wide distribution and being significantly different from ESP and ETH.

The beta diversity was assessed according to the Bray–Curtis dissimilarity index and analyzed with PCoA. The plot in Figure 2B displays the two most informative dimensions of the PCoA space, describing 27.1 and 16.7% of the diversity in the dataset. According to PERMANOVA, the grouping in cohorts was significant (*p* < 0.05), even though extensive overlapping of some cohorts was observed (e.g., ESP and USA). The CHN and ETH cohorts were separated along with PCo1, lying mostly at positive and negative values, respectively. Subjects belonging to the ESP, SWE, and USA cohorts mostly lie at positive PCo2 values, unlike the ones belonging to CHN and ETH, mostly located at negative PCo2.

The genus *Bacteroides* mainly contributed to PCo1 positive values that characterized the CHN subjects (Figure 2C), according to the prevalence of Bacteroidetes over Firmicutes in this cohort (Figure 2A). On the other side, *Prevotella* negatively contributed to the PCo1 autovector. Along with PCo2, the main positive contribution came from Clostridiales, while a negative one came from *Prevotella* and *Bacteroides*.

### β-Glucuronidases Types and Categories

**FIGURE 3:**
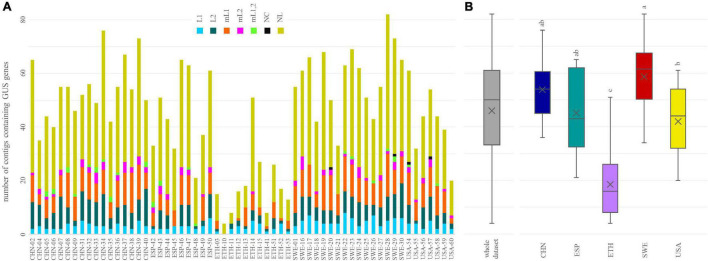
The number of β-glucuronidases (GUS) identified in each metagenome **(A)** and in the whole dataset and in each cohort **(B)**. In **(B)**, cohorts sharing the same letter did not significantly differ (*P* ≥ 0.05, ANOVA, Tukey).

tBLASTn search within the whole sets of contigs pinpointed 218 of the 279 GUS sequences of the inventory of Pollet et al. (2017). Each metagenome encompassed 4 to 82 contigs containing at least a GUS sequence (Figure 3A). The number of different GUS types per subject ranged from 4 (ETH-10) to 70 (SWE-28), with a mean of 40. The richness in different GUS was similar among the cohorts (*p* < 0.05), except in ETH subjects, which presented significantly lower values (*p* < 0.05) (Figure 3B).

Genes encoding GUS of the categories NL, mL1, L2, and L1 were found in all or the vast majority of the metagenomes (≥57), while mL2, mL1,2, and NC GUS genes occurred less frequently (43, 21, and 5 metagenomes, respectively). In terms of both the overall number of sequences and the number of sequences per sample, NL was the richest category, followed by mL1, L2, and L1 (Figure 3A). NL accounted for 129 of the 218 sequences, reaching up to 50 different sequences per sample, while mL1, L2, and L1 respectively accounted for 33, 30, and 13 different sequences and reached up to 16, 14, and 13 sequences per sample. mL2, mL1,2, and NC were represented only by 7, 4, and 2 different sequences, respectively. Despite the different distribution of structural categories among the subjects, the grouping in cohorts was not significant (*p* > 0.05, ANOVA).

**FIGURE 4:**
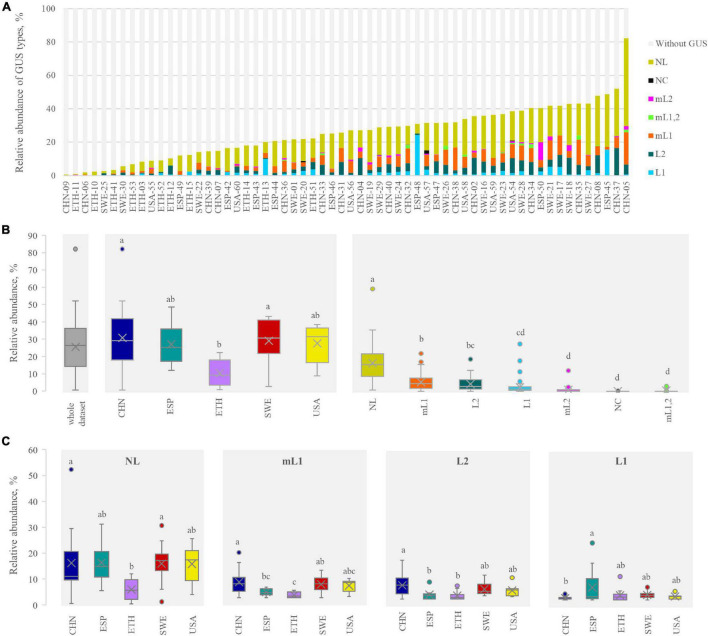
β-glucuronidases (GUS) abundance profile in each metagenome **(A)** and in the whole dataset, in each cohort, and for each structural category **(B)**. The abundance of the four main categories in each cohort is shown in **(C)**. In **(B**,**C)** cohorts or categories sharing the same letter did not significantly differ (*P* ≥ 0.05, ANOVA, Tukey). In determining the GUS abundance profile, for the bins bearing more than one GUS, the abundance was multiplied by the number of GUS therein identified.

The relative abundance of the intestinal bacteria harboring at least a GUS gene was calculated, linking each GUS gene with the relative abundance of the corresponding bin, in its turn obtained by the number of reads mapping in the bin. GUS-encoding bacteria ranged from 0.7% (CHN-09) to 82.2% (CHN-05) (Figure 4A), with a mean abundance of 25.7%. The bacteria harboring GUS genes were significantly less abundant in the ETH than in the other cohorts (*p* < 0.05) (Figure 4B). However, the dataset presented a high variability, even within the same cohort. For instance, the CHN cohort encompassed both subjects where GUS-encoding bacteria presented the lowest and the highest abundance (CHN-9 and CHN-05, respectively).

With regard to the relative abundance of the GUS-encoding bacteria, NL was the most represented, followed by mL1, L2, and L1 (on average, 14.4, 4.6, 3.7, and 2.1%, respectively). Bacteria harboring GUS genes of mL2, NC, and mL1,2 categories were less abundant, accounting on average for less than 1%. Despite the low mean abundance of bacteria harboring L1 genes, this class of GUS presented the highest variability, with encoding bacteria ranging from 0 to 24.3%. The abundance of bacteria encoding each structural category of GUS was similar among the cohorts, with the sole exceptions of NL and mL1, which were less abundant in the ETH cohort (*p* < 0.05, ANOVA, Tukey) (Figure 4C).

### Contribution of the Taxa to β-Glucuronidases Abundance

Abundances, frequencies, and taxa mostly contributing to GUS profile were explored. Taxonomic assignment of GUS was done according to the GUS types classified by Pollet et al. (2017). The abundance of taxa encoding each GUS type was calculated by summing the relative abundance of each bin harboring at least one GUS gene, normalized among the whole set of bins, encompassing or not the GUS genes.

**FIGURE 5:**
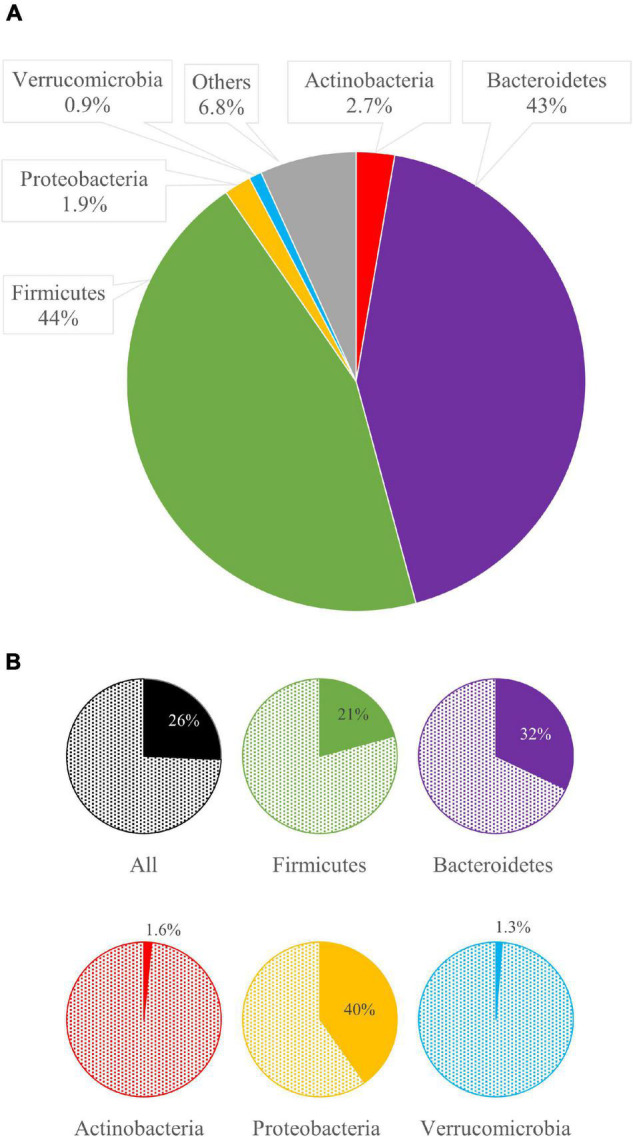
The mean relative abundance of bacterial phyla **(A)** and, for each phylum, the mean abundance of bacteria bearing β-glucuronidases (GUS) genes **(B)**.

Among the 60 metagenomes, 25.7% of the bins encoded for at least one GUS gene. The phyla Bacteroidetes and Firmicutes dominated all the microbiomes and encompassed many bacterial species encoding for at least one GUS gene (32.0 and 20.8%, respectively, Figures 2A, 5).

The genus *Bacteroides* was the main contributor of the intestinal GUS pool, encoding 120 of the 218 GUS identified in this study. Among the 20 most relevant GUS sequences (Table 1), 13 were from *Bacteroides*, including the three most abundant ones (i.e., no. NL-11, mL1-176, and NL-36), which were also the most frequently occurring in the metagenomes. In particular, *Bacteroides* spp. encoding NL-11 reached up to 8.8% in CHN-37 and occurred in 42 metagenomes (Table 1). *Bacteroides vulgatus* encoding mL1-176 was the second most abundant GUS-encoding bin, accounting for 5.3% of total bacteria and being detected in 36 out of 60 metagenomes. *B. dorei* encoding mL1-177 occurred in 13 subjects with a mean abundance of 1.8% and reached more than 10% in CHN-05.

**TABLE 1:** List of the main β-glucuronidases (GUS) sequences identified.

GUS ID	Type	Origin	Mean%	Max% (subject)	Frequency, no. subjects (%)
11	NL	*Bacteroides* spp.	5.6	8.8 (CHN-37)	42 (70)
176	mL1	*Bacteroides vulgatus*	5.3	6.0 (CHN-35)	36 (60)
36	NL	*Bacteroides uniformis*	4.4	8.8 (CHN-37)	30 (50)
220	L1	*Escherichia coli*	3.3	23.3 (ESP-48)	11 (18)
87	NL	*Bacteroides dorei*	2.4	8.8 (CHN-05)	23 (38)
17	NL	*Bacteroides uniformis*	2.4	4.0 (SWE-16)	27 (45)
242	L2	*Bacteroides uniformis*	2.2	5.7 (CHN-08)	22 (37)
177	mL1	*Bacteroides dorei*	1.8	10.6 (CHN-05)	13 (22)
47	NL	*Bacteroides massiliensis*	1.8	4.9 (CHN-35)	16 (27)
67	NL	*Faecalibacterium prausnitzii*	1.7	2.1 (ESP-42)	37 (62)
35	NL	*Bacteroides uniformis*	1.6	2.8 (SWE-21)	15 (25)
223	L1	*Faecalibacterium prausnitzii*	1.6	2.6 (SWE-21)	37 (62)
10	NL	*Bacteroides ovatus*	1.6	2.3 (SWE-26)	25 (42)
185	mL1	*Bacteroides ovatus*	1.6	4.6 (CHN-05)	22 (37)
173	mL1	*Bacteroides massiliensis*	1.5	4.1 (CHN-38)	17 (28)
257	L2	*Bacteroides ovatus*	1.5	2.3 (SWE-26)	26 (43)
53	NL	*Parabacteroides merdae*	1.5	2.5 (CHN-08)	31 (52)
76	NL	*Eubacterium* sp. CAG:180	1.5	14.2 (ESP-45)	14 (23)
126	NL	*Firmicutes*	1.4	2.2 (ESP-47)	36 (60)
134	NL	*Firmicutes*	1.3	2.2 (ESP-47)	34 (57)
180	mL1	*Parabacteroides merdae*	1.3	2.5 (CHN-08)	26 (43)
261	L2	*Bacteroides cellulosilyticus* CAG:158	1.2	4.9 (CHN-08)	16 (27)

*The sequences reported represent the 10 GUS with higher mean abundance, abundance in single microbiome, and frequency in the set of GUS-encoding bacteria.*

Among Firmicutes, the genus *Faecalibacterium* was a major contributor to the abundance of GUS genes. As a whole, the metagenomes encompassed 44 bins of *F. prausnitzii* and 197 bins of *Faecalibacterium* sp., accounting together, on average, for 6.2% of the microbiome, 2.9% encoding at least one GUS gene. In particular, among the 241 bins of *Faecalibacterium*, 125 harbored at least one GUS sequence, mainly belonging to categories L1, NL, or, more rarely, mL1. Eighty-three bins harbored L1 GUS genes, accounting, as a whole, for 0.55% of the metagenomes. In some cases, two diverse GUS were found in the same bin, generally L1 and NL. GUS sequences L1-223 and NL-67 of *Faecalibacterium prausnitzii* were frequently encountered, both being present in 37 of the 60 metagenomes. *F. prausnitzii* encoding GUS L1-223 and GUS NL-67 presented the highest abundance in SWE-21 (2.7%) and in ESP-42 (2.1%), respectively. Other GUS originating from Firmicutes came from the Clostridiales *Eubacterium*, *Ruminococcus*, *Roseburia*, and *Fusicatenibacter* or from unidentified Firmicutes. *Eubacterium* sp. CAG:180 encoding GUS NL-76 occurred in 14 metagenomes and was remarkably abundant in ESP-45 (14.2%) (Supplementary Material 2 and Supplementary Figure 5).

Actinobacteria and Proteobacteria were minor phyla within the analyzed metagenomes, which scarcely contributed to the pool of GUS, since only a minority of bacteria ascribed to these phyla-encoded GUS (1.6 and 1.3%, respectively). Among Proteobacteria, *E. coli* encoding GUS L1-220 mostly participated in GUSome, being detected in 11 metagenomes, remarkably abundant in ESP-48 (23.3%), ESP-45 (12.8%), and ETH-13 (8.5%).

The 40.2% of Verrucomicrobia harbored GUS genes (Figure 5). However, bacteria ascribed to this phylum represented only 0.9% within the set of microbiomes. *Akkermansia* was the main GUS-encoding genus ascribed to this phylum. Among the whole dataset, 13 bins were ascribed to *Akkermansia* sp., accounting for 0.6%, 10 of which harbored GUS genes. In particular, the sequence of GUS mL2-218 was identified in eight bins of *Akkermansia muciniphila*, representing 0.4% of the whole dataset, whereas another two bins of *Akkermansia* encoded the gene mL2-209. A remarkably high amount of *A. muciniphila* encoding GUS mL2-218 (10.6%) was found only in subject ESP-50.

The abundance of specific species known to encode several deeply characterized L1-GUS, such as *C. perfringens*, *E. eligens*, *L. rhamnosus*, *R. gnavus*, *S. agalactiae*, *B. uniformis*, *B. ovatus*, *B. dorei*, *B. fragilis*, and *P. merdae*, was inferred by MetaPhlAn2 metagenomic analysis because CAT/BAT failed to name these bins with a species designation. *L. rhamnosus*, *S. agalactiae*, and *C. perfringens* lie below the limit of detection in most of the metagenomes (≥ 53/60) and, when found, were present at very low abundances (≤ 0.15%). *E. eligens* and *R. gnavus* were more represented (observed in 51 and 39 subjects, respectively) with a mean abundance of 1.0% and 0.3%. *B. uniformis* was the most abundant among the above-mentioned Bacteroidetes, being detected in 55/60 metagenomes at the mean abundance of 2.7%, followed by *B. ovatus* (1.1%; 51/60), *B. dorei* (1.0%; 51/60), and *B. fragilis* (0.5%, 32/60), while *P. merdae* was pinpointed in 47 subjects with a mean abundance of 1.2%.

**FIGURE 6:**
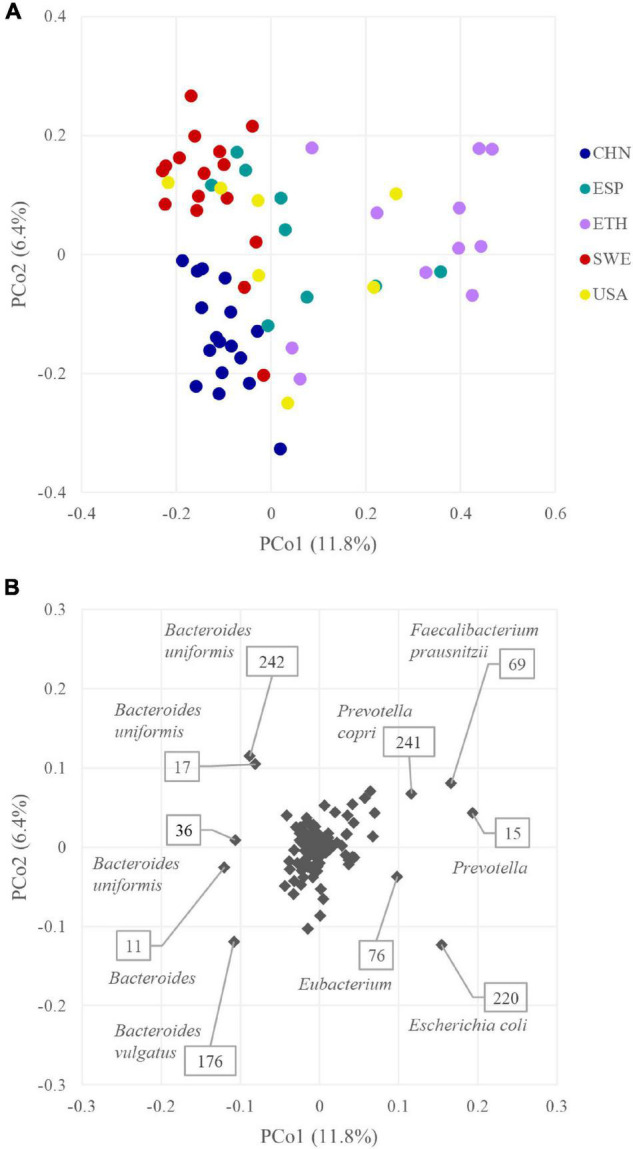
**(A)** PCoA plot of beta diversity based on Jaccard dissimilarity index of the β-glucuronidases (GUS) profiles of the 60 metagenomes. **(B)** PCoA plot of the GUS contribution to GUSome differentiation.

The beta diversity was computed based on the presence of the 218 GUS sequences. Jaccard metrics were utilized, with a qualitative approach to prevent abundant *Bacteroides* from concealing the differences among metagenomes and cohorts. The plot in Figure 6A displays the PCoA space of beta diversity in the two most informative dimensions, describing 11.8 and 6.4% of the diversity. Cohort grouping based on the presence of GUS sequences was statistically significant according to PERMANOVA (*p* < 0.05). The CHN and SWE cohorts were characterized by a negative value of PCo1, and the ETH by a positive one. Most of the GUS that negatively contributed to PCo1 originated from *Bacteroides*, mainly *B. uniformis* and *B. vulgatus*. On the other side, the main positive contributors to PCo1 were GUS sequences from *Prevotella*, *Prevotella copri*, *F. prausnitzii*, *Eubacterium*, and *E. coli* (Figure 6B).

## Discussion

Pharmacomicrobiomics investigates the interplay of microbiome diversity and drug disposition and response and may provide an important basis in personalized medicine (Doestzada et al., 2018; Hassan et al., 2021). The role of bacterial GUS on drug bioavailability and biological effects (i.e., the reactivation and absorption vs the excretion) encouraged our deep analysis of interindividual variability of the intestinal GUS, originating from different microbiome compositions. With this aim, metagenome data of 60 healthy adults from different geographic provenance were utilized to predict and compare their GUSome, i.e., the abundance and the diversity of GUS genes and structural categories.

The abundance of intestinal bacteria harboring at least a GUS gene was highly variable among subjects, ranging from a very small minority (e.g., 0.7% in CHN-09) to an overwhelming majority (e.g., 82.2% in CHN-05) of the intestinal microbial community. No significant difference between cohorts could be identified, except for ETH, where GUS-encoding bacteria were significantly less abundant. The GUSome (i.e., the abundance and the diversity of GUS genes within the metagenome) was generally dominated by category NL, followed by mL1, L2, and L1, in agreement with literature information (Pollet et al., 2017). In some subjects, particularly in the ETH cohort, a low level of GUS sequences was in relation to the low abundance of bacteria encoding NL and mL1 GUS.

NL and L2 GUS are involved in processing commonly available large substrates (e.g., mucins, glycosaminoglycans, plant polysaccharides, etc.) (Pollet et al., 2017). On the one hand, their abundance and frequency in the microbiome are presumably related to a role in the energetic metabolism of intestinal bacteria and may be a positive adaptive trait related to the evolution of bacteria in the colonic environment. On the other hand, GUS belonging to categories L1 and mL1 are involved in the hydrolysis of small molecules, including drugs (Pollet et al., 2017; Biernat et al., 2019).

L1 GUS process estrone and estradiol glucuronides, affecting the estrogen profile and promoting the onset of hormonal disorders (Baker et al., 2017; Ervin et al., 2019). L1 GUS also participate in the toxicity of irinotecan since they are key effectors of metabolite SN-38 reactivation and have been targeted by specific inhibitors to protect the intestinal epithelial cells and to reduce chemotherapy-induced diarrhea (Bhatt et al., 2020; Jariwala et al., 2020; Parvez et al., 2021; Wang et al., 2021). Moreover, L1 GUS are responsible for the deconjugation of glucuronidated non-steroidal anti-inflammatory drugs (NSAIDs), which is among the most commonly used medications worldwide (Maseda and Ricciotti, 2020).

In most of the microbiomes analyzed in this study, the bacteria encoding L1 GUS genes presented a relatively low abundance. The ratio of bacteria encoding L1 genes was less than 2% in 34 subjects and accounted for 2–5% in 12, resulting significantly higher in the SPA cohort than in the CHN one. Three microbiomes presented a very high abundance of L1 GUS-encoding bacteria (ESP-48 24.3%, ESP-45 15.6%, and ETH-13 9.9%). The most relevant L1 GUS, in terms of frequency and abundance of the corresponding bins, were L1-220 GUS of *E. coli* and L1-223 of *F. prausnitzii* (Table 1). *E. coli* containing the L1-220 GUS gene were present in 11 out of 60 metagenomes and generally occurred in low concentrations. These results are consistent with data reported in a recent study aimed to characterize intestinal *E. coli* from healthy adults (Raimondi et al., 2019), revealing that approx. a fifth of *E. coli* isolates did not present β-glucuronidase activity. In the microbiomes where L1-encoding *E. coli* presented a high abundance (e.g., ESP-48 23.3%, ESP-45 12.8%, and ETH-13 8.5%), it is expected that L1 GUS from this commensal pathobiont could heavily interact with drug glucuronides, affecting efficacy and/or toxicity. *Faecalibacterium* sp. and *F. prausnitzii*, which are commensals associated with beneficial health effects, were much more frequent and abundant, representing on average 6.2% of the microbiomes herein analyzed. Despite their abundance, only a negligible ratio of *Faecalibacterium* encoded L1 GUS, accounting for 0.55% of the set of bacteria. However, the small portion of *Faecalibacterium* encoding L1-223 remained the major player in L1-catalyzed deglucuronidations, being identified in the majority of the subjects. On the other side, *E. coli* encoding L1-220 could participate in deconjugation in few microbiomes where it is exceptionally abundant. However, L1-220-encoding *E. coli* lie below the limit of detection in most cases.

Literature reports several L1-GUS that have been deeply characterized in some Firmicutes (i.e., *C. perfringens*, *E. eligens*, *L. rhamnosus, R. gnavus*, and *S. agalactiae*) and Bacteroidetes (i.e., *B. ovatus*, *B. dorei*, *B. fragilis*, and *P. merdae*) (Pollet et al., 2017; Biernat et al., 2019; Jariwala et al., 2020). CAT/BAT did not pinpoint nearly any bin of these species, likely because of the high fragmentation of metagenomic assemblies; thus, they were searched using MetaPhlAn2, which allowed higher accuracy of taxonomic profiling. *C. perfringens*, *L. rhamnosus*, and *S. agalactiae* were found in a minority of metagenomes at a negligible concentration. On the other hand, *E. eligens* and *R. gnavus* occurred more frequently and abundantly in the metagenomes and thus are expected to contribute to the pool of L1 GUS.

L1 GUS are involved in the deconjugation of glucuronidated NSAIDs, such as diclofenac. The glucuronide of diclofenac is synthesized in the liver and excreted in the gut lumen (Maseda and Ricciotti, 2020). L1 GUS from *E. eligens* is the most active in hydrolyzing this glucuronide (kcat 138 s^–1^), followed by the corresponding of *S. agalactiae*, *C. perfingens*, *F. prausnitzii*, *E. coli*, and *R. gnavus* (kcat from 97 to 30.7 s^–1^), while that from *L. rhamnosus* presents a catalytic efficiency approx. one magnitude lower (Biernat et al., 2019). Taking into account the abundance of these species, *E. eligens*, *F. prausnitzii*, and *E. coli* are expected to mostly contribute to the release of the diclofenac aglycone from the glucuronide. The peculiar richness of some of these species, as detected in our dataset for *E. coli*, likely modifies the clearance of the drug, facilitating reuptake and recirculation. A similar pattern of catalytic activity has also been assessed for SN-38, with L1 GUS reactivating this toxic metabolite of the anticancer drug irinotecan and causing consequent gastrointestinal toxicity (Jariwala et al., 2020; Parvez et al., 2021).

Glucuronides of small drugs can be also hydrolyzed by mL1 GUS (Wallace et al., 2015; Biernat et al., 2019). For instance, GUS mL1-188 of *B. fragilis* was described to possess a remarkably high activity against p-nitrophenol-β-D-glucuronide, diclofenac-glucuronide, and SN38-glucuronide, albeit lower than L1 enzymes (Biernat et al., 2019; Bhatt et al., 2020). In the microbiomes analyzed in this study, the abundance of mL1 GUS-encoding bacteria was significantly higher than L1, with the CHN cohort significantly richer than the ESP and ETH ones. Among the 16 mL1 GUS sequences herein identified, the one encoded by *B. vulgatus* (mL1-176) was the main in terms of abundance and frequency, followed by those encoded by *B. dorei*, *B. ovatus*, and *B. massiliensis* (mL1-177, mL1-185, and mL1-173, respectively). mL1 GUS 188 of *B. fragilis*, the structure and activity of which has been deeply characterized (Wallace et al., 2015; Biernat et al., 2019), was retrieved in 13 out of 60 metagenomes, but its mean abundance was 0.15%. Since the genus *Bacteroides* generally occurs at high levels in the microbiomes and encodes the main mL1 GUS, it is conceivable that this genus plays an important role in the regeneration of parent compounds or active metabolites in the gut, evoking their major contribution to drug reactivation.

Within the observed broad interindividual heterogeneity of GUS profiles, the described differences in terms of L1 and mL1 GUS likely represent the major drivers of the variability in pharmacomicrobiomics, affecting the level of active deconjugated molecules and thus influencing drug response and toxicity. Strains belonging to the species *B. ovatus*, *B. dorei*, *B. fragilis*, *E. coli*, *E. eligens*, *F. prausnitzii*, *P. merdae*, and *R. gnavus* emerged as pivotal in the differentiation of catalytic activity toward small glucuronides in the host and can be claimed as the main players in the reactivation of drug metabolites by the various intestinal microbial ecosystems. The targeted control of gut microbiota, by modulating metabolic diversity, represents a future perspective to govern and reduce the deconjugation activity. This strategy, also based on the administration of selected probiotics, could mitigate extreme and severe differences among patients in terms of adverse drug responses, responsible for numerous disease states.

## Data Availability Statement

The original contributions presented in the study are included in the article/Supplementary Material, further inquiries can be directed to the corresponding author.

## Author Contributions

FC, AA, SR, and MR conceived the study. FC, EM, and RR carried out the bioinformatic analysis. AA, SR, FC, and AZ conducted data interpretation and presentation. FC and MR wrote the manuscript with contributions from SR, RR, EM, AZ, and AA. All authors contributed to the article and approved the submitted version.

## Conflict of Interest

The authors declare that the research was conducted in the absence of any commercial or financial relationships that could be construed as a potential conflict of interest.

## Publisher’s Note

All claims expressed in this article are solely those of the authors and do not necessarily represent those of their affiliated organizations, or those of the publisher, the editors and the reviewers. Any product that may be evaluated in this article, or claim that may be made by its manufacturer, is not guaranteed or endorsed by the publisher.

## Footnotes

1
https://usegalaxy.eu
