# Supplementary material for: β-Glucuronidase Pattern Predicted From Gut Metagenomes Indicates Potentially Diversified Pharmacomicrobiomics
Source: Front Microbiol. 2022 Mar 3;13:826994. doi: 10.3389/fmicb.2022.826994 (PMC8928169; doi:10.3389/fmicb.2022.826994)
Supplement: Supplementary file 3 [file Data_Sheet_3.pdf]

β-glucuronidase pattern predicted from gut metagenomes indicates potentially diversified pharmacomicrobiomics

Francesco Candeliere[a], Stefano Raimondi[a], Raffaella Ranieri[a], Eliana Musmeci[a], Alfonso Zambon[b], Alberto Amaretti[a,c], Maddalena Rossi[a,c,*]

[a] Department of Life Sciences, University of Modena and Reggio Emilia, Modena, Italy

[b] Department of Chemistry and Geological Sciences, University of Modena and Reggio Emilia, Modena, Italy

[c] Biogest-Siteia, University of Modena and Reggio Emilia, Modena, Reggio Emilia, Italy

*** Correspondance:**

maddalena.rossi@unimore.it

Supplementary Material

Supplementary Table S1. Accession numbers of the 60 metagenomes utilized in this work. Number and length of reads are reported. First three letters in sample's name indicate one of the five cohorts: China (CHN), Ethiopia (ETH), Spain (ESP), Sweden (SWE), and United States of America (USA).

Sample	Accession number	Reads length	N° reads
SWE-01	ERS554193	100	1.27E+08
CHN-02	SRR10680552	150	2.55E+07
ETH-03	SRR8784372	100	3.43E+07
CHN-04	SRR10680551	150	2.59E+07
CHN-05	SRR10680550	150	2.47E+07
CHN-06	SRR10680549	150	2.74E+07
CHN-07	SRR10680548	150	2.66E+07
CHN-08	SRR10680547	150	3.03E+07
CHN-09	SRR10680545	150	2.90E+07
ETH-10	SRR8784387	100	1.94E+07
ETH-11	SRR8784374	100	2.35E+07
ETH-12	SRR8784376	100	2.99E+07
ETH-13	SRR8784385	100	3.02E+07
ETH-14	SRR8784383	100	4.57E+07
ETH-15	SRR8784379	100	2.50E+07

Sample	Accession number	Reads length	N° reads
SWE-16	ERR636369	100	9.12E+07
SWE-17	ERR636383	100	7.10E+07
SWE-18	ERR636385	100	1.03E+08
SWE-19	ERR636375	100	1.78E+08
SWE-20	ERR636405	100	8.48E+07
SWE-21	ERR636371	100	9.41E+07
SWE-22	ERR636411	100	8.67E+07
SWE-23	ERR636351	100	5.67E+07
SWE-24	ERR636373	100	7.78E+07
SWE-25	ERR636353	100	1.12E+08
SWE-26	ERR636391	100	6.57E+07
SWE-27	ERR636389	100	9.99E+07
SWE-28	ERR636355	100	1.01E+08
SWE-29	ERR636359	100	8.42E+07
SWE-30	ERR636363	100	9.76E+07
CHN-31	SRR10680544	150	2.51E+07
CHN-32	SRR10680543	150	2.93E+07
CHN-33	SRR10680542	150	2.76E+07
CHN-34	SRR10680541	150	2.73E+07
CHN-35	SRR10680540	150	2.49E+07
CHN-36	SRR10680445	150	2.50E+07
CHN-37	SRR10680443	150	2.73E+07
CHN-38	SRR10680442	150	2.73E+07
CHN-39	SRR10680441	150	2.49E+07
CHN-40	SRR10680439	150	2.50E+07
ETH-41	SRR8784390	100	2.50E+07
ESP-42	ERR3452699	150	2.31E+07
ESP-43	ERR3452574	150	3.12E+07
ESP-44	ERR3452529	150	3.37E+07
ESP-45	ERR3452318	150	3.14E+07
ESP-46	ERR3451635	150	3.60E+07
ESP-47	ERR3450606	150	3.45E+07
ESP-48	ERR3450296	150	2.54E+07
ESP-49	ERR3450229	150	4.47E+07
ESP-50	ERR3450203	150	2.72E+07
ETH-51	SRR8784391	100	2.28E+07
ETH-52	SRR8784395	100	2.42E+07
ETH-53	SRR8784394	100	1.72E+07
USA-54	ERR2641799	150	1.37E+07
USA-55	ERR2641792	150	9.17E+06
USA-56	ERR2641793	150	9.40E+06
USA-57	ERR2641795	150	1.22E+07
USA-58	ERR2641798	150	1.92E+07
USA-59	ERR2641800	150	1.11E+07

Sample	Accession number	Reads length	N° reads
USA-60	ERR2641801	150	1.04E+07

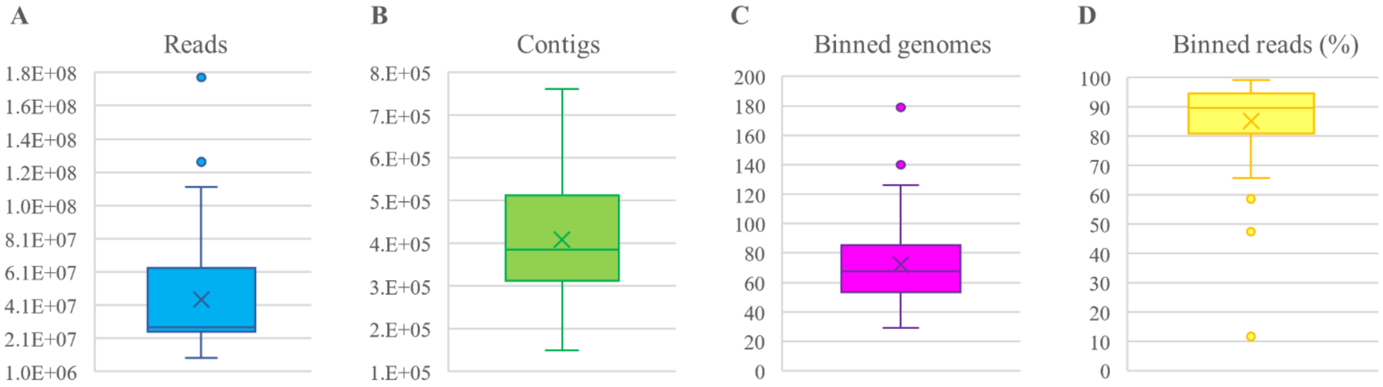

Supplementary Figure S1. Metagenomic data of the 60 microbiomes: the mean (X), the median (central line), the 25th and 75th percentiles (colored box), the 10th and 90th percentiles (whiskers), and the outliers (dots) are indicated. A: number of reads; B: number of contigs obtained by metaSPAdes assembly; C: number of bins reconstructed by MaxBin2; D: reads mapped on the binned contigs by Bowtie2 (%).

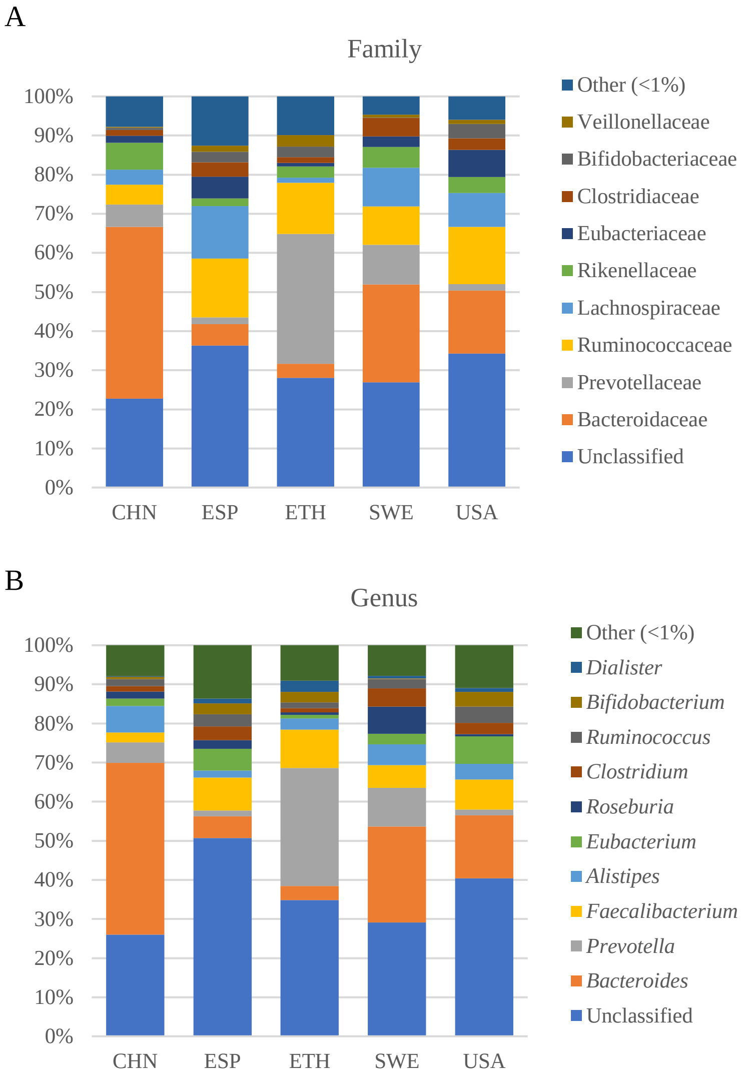

Supplementary Figure S2. Mean relative abundance of the main families (A) and genera (B) identified in the whole dataset and in the five cohorts.

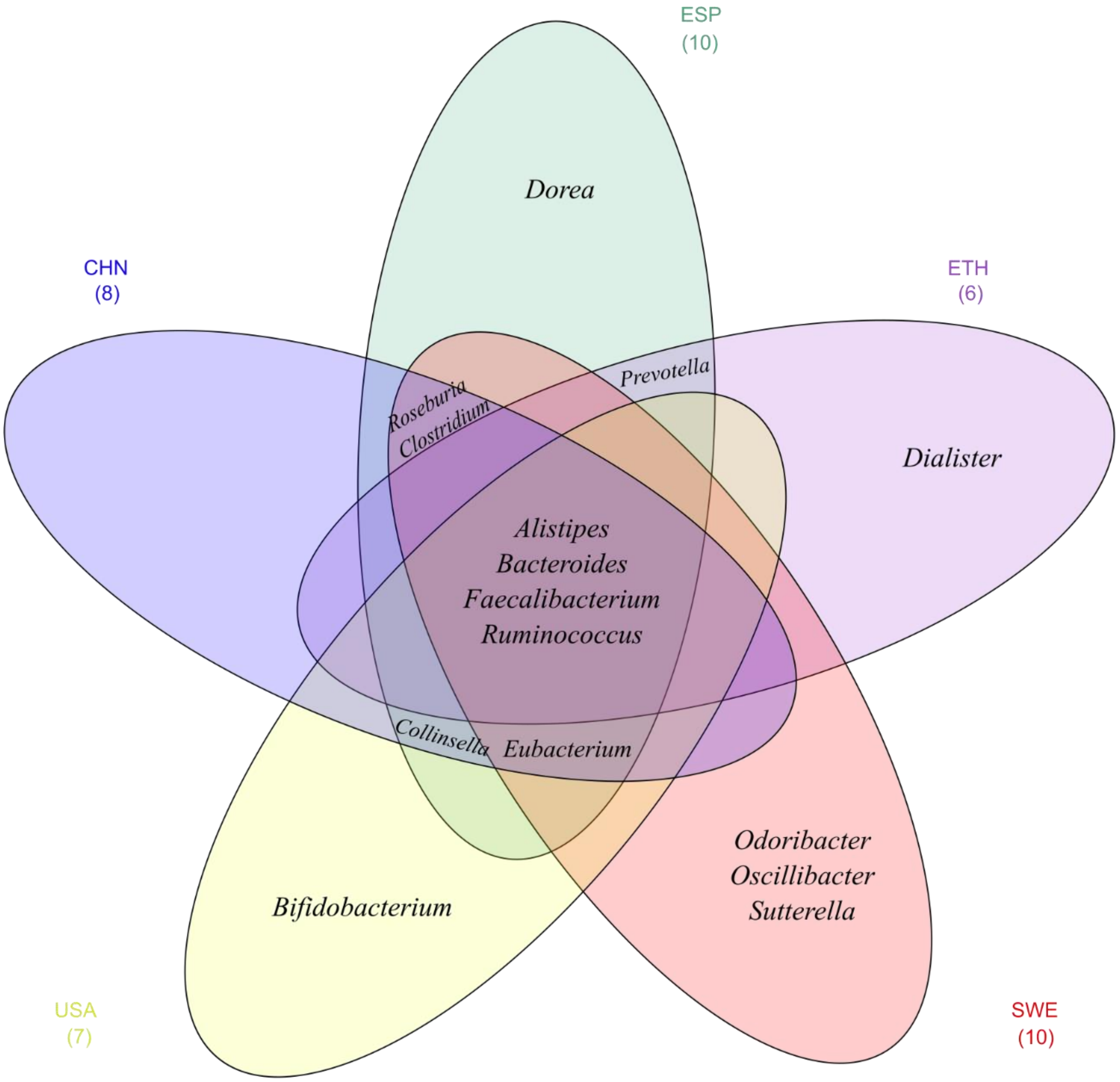

Supplementary Figure S3. Venn's diagram of core genera identified in each cohort.

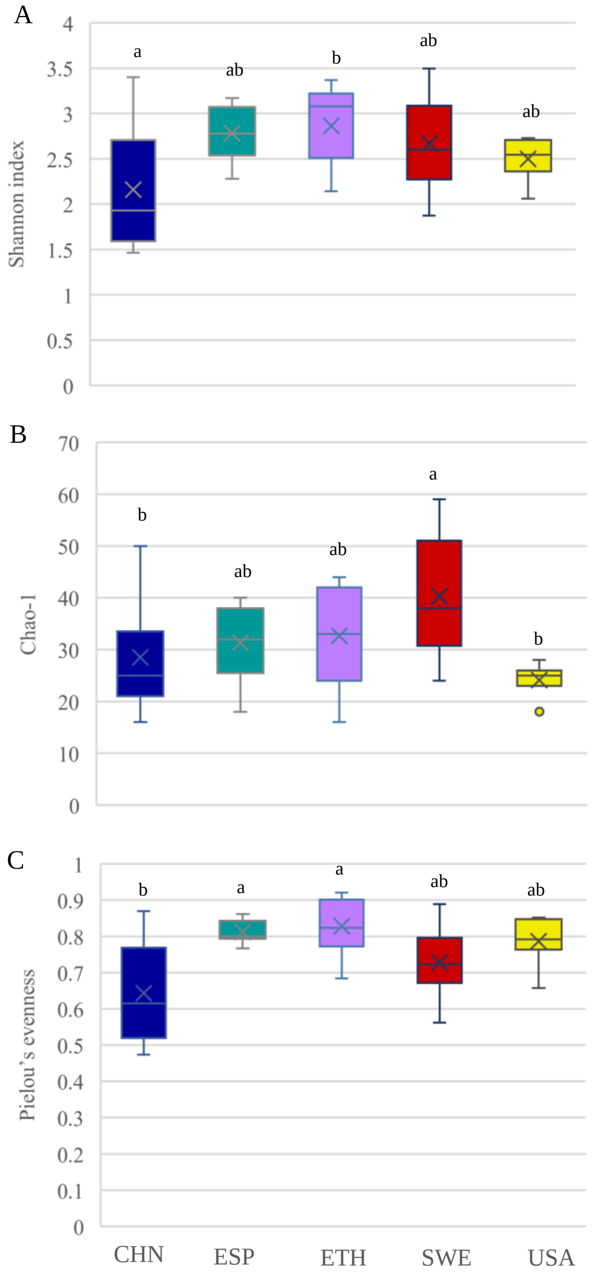

Supplementary Figure S4. Alpha diversity based on Shannon index (A), Chao-1 index (B) and Pielou's evenness (C). Cohorts sharing the same letter did not significantly differ ($P \geq 0.05$, Kruskall-Wallis, Dunn).

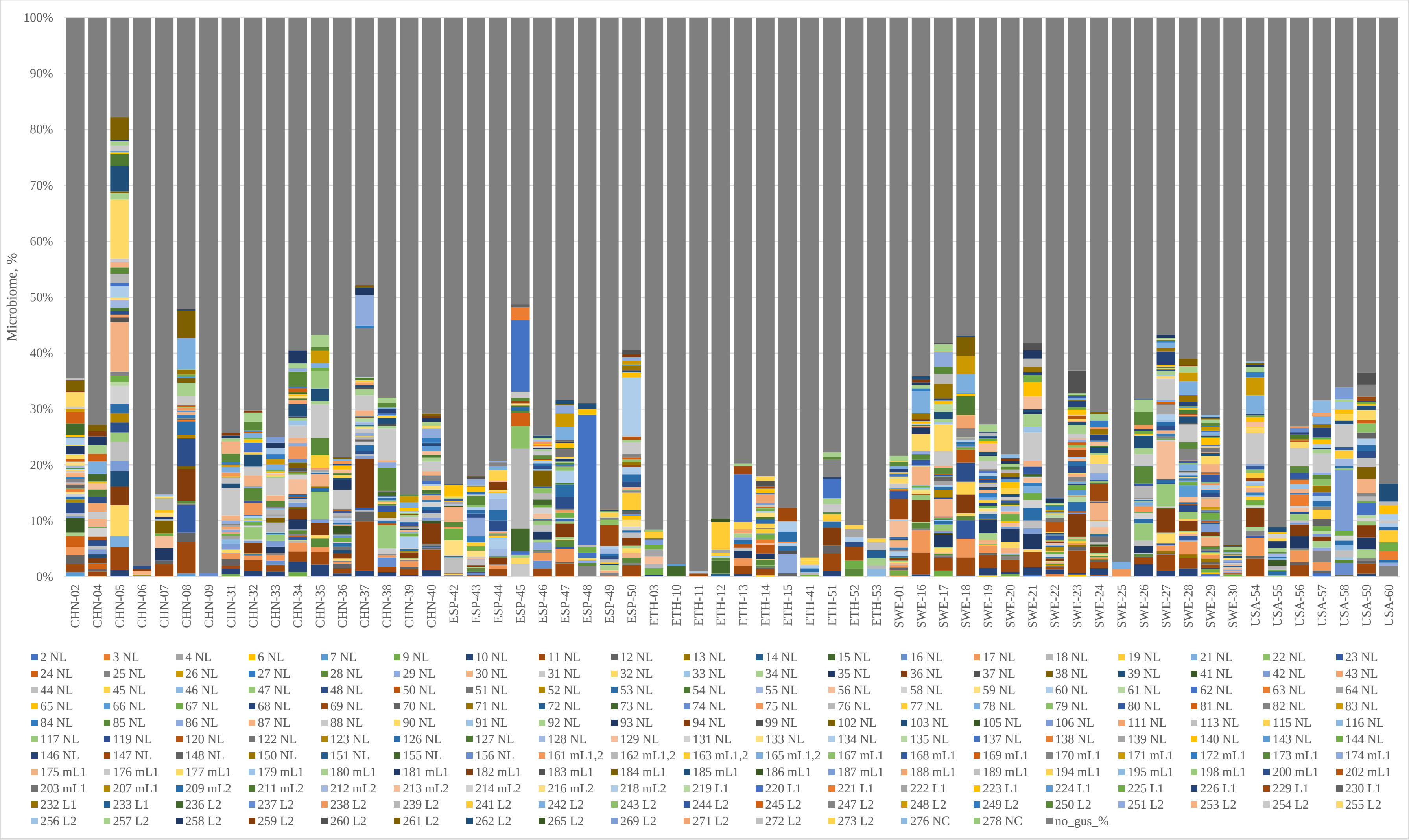

Supplementary Figure S5. Distribution of GUS sequences in the microbiome of 60 subjects. The name of the species corresponding to each sequence can be found in Supplementary Datasheet S2
